# A New Departure

**Published:** 1883-01

**Authors:** 


### A New Departure.

[Read before the Mad River Dental Society, Oct. 24, 1882, By Wm. A. Pease.]

One of the greatest obstacles to dental success, and one that causes, perhaps, the greatest perplexity and trouble; by limiting efforts to be useful, and at the same time causing the patient much pain and a disbelief in the ability of dentists to save the teeth, is the formation of a sack at the end of a root.

Whenever and from whatever cause a pulp dies decomposition must take place, in obedience to a law of Nature that is imperative, which can be only partially and temporarily thwarted by any means man has hitherto discovered. It has been said that Nature abhors a vacuum; and it may be said with equal truth that she abhors undecomposed dead animal or vegetable matter. She is a utilitarion, husbanding her resources, metamorphosing them, always with a view to utility and profit. Her sympathies are with life, not with death, and her efforts are always directed to give it a generous support, by presenting it ready formed, with a rich assimilable material. Hence, there is no new creation of matter; and no accumulation of old, beyond her present, or prospective wants. Nothing is worn—effete; or passes beyond that form, which underlies utility. Capabilities are lost and acquired; matter pursues an endless circuit; sometimes as erratic as that of a comet; but once having become organic, it always returns to the initial point of capability of assimilation by some organization.

In view of this law of Nature the question arises—have not our labors been in the wrong direction, in trying to thwart the efforts of Nature, instead of furthering them, and accelerating them by all the means at our command. We have tried to arrest decomposition long enough to know that all of our efforts are futile, Nature will triumph in spite of carbolic acid, naptha, or wood creosote, arsenious acid, or the salts of soda. They are simply retarders of, or more or less arrestors of combustion, dependant on temperature, moisture, and the more or less abundant supply of oxygen. High temperature accompanied with moisture accelerates it; but a high and dry temperature, notwithstanding abundance of oxygen retards it. Thus meat when exposed to the air in Colerado, or New Mexico, does not readily putrify; and some of the mummies of Egypt have lasted four thousand years, which could not have been in that hot climate had there been abundance of moisture, notwithstanding the interposition of non-oxidziable materials, as sodium chloride, and an almost impenetrable wrapping, to oxygen, of a carbolized coated and cemented fabric. It requires a product of combustion to arrest combustion, or rather a material contained in a combustible one, that is incombustible at ordinary temperatures, rejected, set free during combustion, such as soot. That in any of its various forms is only a retarder, as in the presence of heat and moisture oxygen will ultimately overcome it. Alternations of heat or cold; of dryness or moisture, by giving periods of rest, are unfavorable to oxydation; hence in a temperate climate specimens of animal matter can be preserved for a considerable time. But soot, or its equivalent, is only a retarder; as, in the presence of heat and moisture oxygen will ultimately overcome it. The best salted, and carbolized ham must be kept dry or oxygen will ultimately destroy it; and the most thoroughly carbolized and arsenicated pulp, though protected in its chamber from oxygen by the best filling a dentist can insert, will decompose and set free an expansible gas. If the volume of that gas were no greater than the material from which it is evolved no harm would be done, as it would not, then, press through the foramen upon the unyielding base of the socket to overcome the resistance of both socket and connecting tissue, and elevate the tooth. It is the pressure of that gas that compresses the remains of the pulp to the filmy fibers we sometimes see before it has been wholly consumed.

It is hardly pertinent to the present purpose to inquire how oxygen gains access to the pulp, and thus liberates a gas; unless to show the persistency of Nature to accomplish the complete combustion of all dead animal matter. A defective filling is no answer, as precisely the same result is attained in a sound tooth. The process may be slower, but it is sure. There is little probability that oxygen penetrated through the walls of the tooth and it is as improbable that the affinity of the dead pulp abstracted it from its bony encasement and thus partially decomposed it. That oxygen must come from the blood, and leave it deoxidized to that extent. It is within the purview of Nature, in rendering all animal matter inert; that a dead pulp shall have so great affinity for oxygen as to draw it from the blood and overcome its affinity for it. But how is that done? At the separation of the living parts from the dead pulp, what ensues? Do they remain in contact, amicably side by side; or does the effort to accommodate the now superfluous blood by anastomosis cause a fullness of the part, that shall press upon the dead pulp, and thus facilitate absorption of oxygen from the blood, and whatever is absorbable in the blood from it, or does the non-palpitant dead pulp recede? It is probable that is does, as a cadaver is undoubtedly smaller before inflation by decomposition takes place than in life. There are many other phenomena attendant upon the simple, and as once thought, unimportant matter of the death of the pulp, that are not necessary to be considered at present. They only show how important the minutest part of a man is to the whole; and what a disturbance its death creates, as well as the labor it entails upon that indefatigable scavenger, oxygen, ever busy running up and down within the maxima and minor cloacoæ of our bodies to free us from impurities, and make us tolerable to ourselves, and presentable to others.

Now, experience has shown that the anti-septic method of treating a dead pulp does not antisept. Probably all of us have seen many times the evidence of this. After having extracted a tooth, in which some part of the pulp has been anti-septically treated, the root having been split open, there is the smell of the antiseptic, showing that its strength has not been exhausted; while there is the smell of the gas and the physical signs of a progressing decomposition. That is to be expected. A piece of a mummy sealed in the chamber of a tooth, in the presence of moisture and animal heat, would undoubtedly decompose. The process might be slow, but it would be sure. What, then, is our remedy?—a new departure. Instead of trying to arrest decomposition, which has been but a partial success—a practical failure; let us try by all the means in our power, chemical or otherwise, to accelerate decomposition; until the smallest remains of a pulp, we cannot remove, has become inert. That may involve treatment—a temporary stopping, perhaps only of cotton saturated with some gum insoluble in the mouth, but it is believed from incomplete experiments that it will be shorter and surer than the present method. What is wanted is exemption from a lingering decomposition, gas —inert matter, silica, alumina, or even humors would cause no irritation could they be substituted for the remains of the pulp. Reduce the pulp quickly and surely to its ultimate constituents and all trouble would end. That reduction would cause shrinkage—the withdrawal of the remains from the live tissue at the end, and without the foramen, and irritation would cease. A cicatrized surface is not fertile in exudations; and it is not probable that they would be poured into the pulp cavity, even though partially empty. But that is not presumable, if all decomposable matter has been decomposed, as it should be, there would be nothing to extract the little oxygen that might remain in the pulp cavity, and a little compressed air would be a bar to exudation.

In the absence of a perfunctory, over-zealous and careless manipulation that presses the debris down upon the live parts, it is believed the chances of irritation would be very small. Thus far my experiments, though extending over more than two years, have been few; as the number of persons who can be seen frequently are not numerous; but they have been of such an encouraging nature as to warrant my laying the matter before you for an expression of opinion. Believing in non-irritants, the injection of water, sometimes quite warm, has been the means I have employed, together with the free use of a slender and flexible probe with which I have pierced and lacerated the remnants of the pulp as much as possible. That gave free access of water and oxygen to all parts of it, and rapidly deprived it of its gas-bearing properties. Once thoroughly decomposed there should be no danger of after trouble, especially if a little solution of morphine or other sedative is carried down with the probe and left as near to the foramen as possible. The tubuli of the dentine have not been filled with any carbolized or acrid substance to encroach upon, and irritate those of the cementum. This method of treatment seems to me more rational—am I in error? As I have said that I do not believe in irritants I do not intend by that to say that other chemical means may not be useful, or even better, but that I have not tried them. Any non-irritant rich in oxygen that is held by a feeble tenure of affinity, may and probably would be useful. But in all experiments it should be borne in mind that the life-like color of the tooth depends upon non-infiltrated tubules, and that the rapid decomposition of the pulp is dearly obtained at the expense of color or a lowered vitality. It is of the utmost importance that the connection of the cementum should be healthy.

Now, gentlemen, these are suggestions that seem plausible to me, but they have not passed much into experiment—they need verification. I leave them with you as it is little likely, that I will be able to work them out to any certain result. If they have a grain of salt that savors of usefulness, opportunity presenting, you can test them; if not, I have occupied your time for no useful purpose.

